# Supplementary material for: Apolipoprotein-E (Apoe) ε4 and cognitive decline over the adult life course
Source: Transl Psychiatry. 2018 Jan 10;8:18. doi: 10.1038/s41398-017-0064-8 (PMC5802532; doi:10.1038/s41398-017-0064-8)
Supplement: Supplementary file 1 — Supplemental Table S1 [file 41398_2017_64_MOESM1_ESM.docx]

|  | Model One (APOE only) | | | | Model Two (APOE + Gender) | | | | Model Three (APOE + Gender + Childhood Cognition) | | | |
| --- | --- | --- | --- | --- | --- | --- | --- | --- | --- | --- | --- | --- |
|  | β | LCI | UCI | p | β | LCI | UCI | p | β | LCI | UCI | p |
| **AGE 43** (n=2183)  No APOE-ε4  Heterozygous APOE-ε4  Homozygous APOE-ε4 | Reference  2.66  3.45 | -4.84  -15.08 | 10.15  21.98 | .75 | Reference  3.87  4.05 | -3.56  -14.29 | 11.30  22.40 | .56 | Reference  3.30  1.93 | -4.09  -16.32 | 10.69  20.19 | .68 |
| BIC | 25169.86 | | | | 25132.73 | | | | 25113.85 | | | |
| **AGE 53**  (n=2347)  No APOE-ε4  Heterozygous APOE-ε4  Homozygous APOE-ε4 | Reference  2.71  3.19 | -4.45  -14.32 | 9.87  20.70 | .73 | Reference  3.74  3.47 | -3.38  -13.91 | 10.85  20.84 | .57 | Reference  3.24  2.04 | -3.82  -15.20 | 10.30  19.28 | .66 |
| BIC | 27017.86 | | | | 26988.16 | | | | 26957 | | | |
| **AGE 60-64**  (n=1694)  No APOE-ε4  Heterozygous APOE-ε4  Homozygous APOE-ε4 | Reference  2.07  -3.37 | -6.04  -23.02 | 10.18  16.28 | .82 | Reference  3.08  -3.19 | -5.03  -22.77 | 11.18  16.40 | .70 | Reference  2.47  -5.26 | -5.53  -24.60 | 10.47  14.08 | .70 |
| BIC | 19348.6 | | | | 19343.11 | | | | 19305.09 | | | |
| **AGE 69** (n=1643)  No APOE-ε4  Heterozygous APOE-ε4  Homozygous APOE-ε4 | Reference  3.12  5.36 | -5.02  -14.75 | 11.26  25.47 | .68 | Reference  3.68  5.66 | -4.44  -14.38 | 11.80  25.69 | .61 | Reference  3.25  3.56 | -4.82  -16.35 | 11.31  23.48 | .71 |
| BIC | 18750.8 | | | | 18745.07 | | | | 18729.14 | | | |

SUPPLEMENTAL DATA

**TABLE S1: CROSS-SECTIONAL RESULTS OF APOE STATUS ON TOTAL TIMED LETTER SEARCH TASK SCORE**

*All figures rounded to 2 decimal places*
