# Supplementary material for: Apolipoprotein-E (Apoe) ε4 and cognitive decline over the adult life course
Source: Transl Psychiatry. 2018 Jan 10;8:18. doi: 10.1038/s41398-017-0064-8 (PMC5802532; doi:10.1038/s41398-017-0064-8)
Supplement: Supplementary file 2 — Supplemental Table S2 [file 41398_2017_64_MOESM2_ESM.docx]

SUPPLEMENTAL DATA

**TABLE S2: LONGITUDINAL RESULTS OF APOE STATUS ON TOTAL TIMED LETTER SEARCH TASK SCORE BETWEEN AGES 43 AND 69**

|  | Model One (APOE only) | | | | Model Two (APOE + Gender) | | | | Model Three (APOE + Gender + Childhood Cognition) | | | |
| --- | --- | --- | --- | --- | --- | --- | --- | --- | --- | --- | --- | --- |
| INTERCEPT TERMS | β | LCI | UCI | p | β | LCI | UCI | p | β | LCI | UCI | p |
| APOE-ε4 Status:  No APOE-ε4  Heterozygous APOE-ε4  Homozygous APOE-ε4  Gender (Female)  Childhood Cognition (8)  SLOPE TERMS  Decline per Year (Linear)  Decline per Year (Quadratic)  APOE-ε4 Status:  No APOE-ε4 Slope  Heterozygous APOE-ε4 Slope  Homozygous APOE-ε4 Slope | Reference  0.79  3.04  **-21.68**  **0.17**  Reference  0.06  -0.03 | -17.35  -41.03  **-23.43**  **0.15**  -0.25  -0.78 | 18.94  47.11  **-19.92**  **0.18**  0.37  0.72 | .93  .89  **<.01**  **<.01**  .72  .94 | Reference  1.71  3.59  **17.63**  **-21.66**  **0.17**  Reference  0.06  -0.03 | -16.35  -40.32  **12.61**  **-23.42**  **0.15**  -0.25  -0.78 | 19.79  47.49  **22.64**  **-19.91**  **0.18**  0.37  0.72 | .85  .87  **<.01**  **<.01**  **<.01**  .72  .94 | Reference  1.08  1.89  **17.31**  **11.39**  **-21.61**  **0.17**  Reference  0.06  -0.03 | -17.00  -42.02  **12.36**  **8.42**  **-23.36**  **0.15**  -0.25  -0.78 | 19.16  45.80  **22.26**  **14.37**  **-19.86**  **0.18**  0.37  0.72 | .91  .93  **<.01**  **<.01**  **<.01**  **<.01**  .72  .94 |
| BIC | 85589.18 | | | | 85550.02 | | | | 85502.04 | | | |

*All figures rounded to 2 decimal places*
